# A New Bearing Fault Diagnosis Method Based on Capsule Network and Markov Transition Field/Gramian Angular Field

**DOI:** 10.3390/s21227762

**Published:** 2021-11-22

**Authors:** Bin Han, Hui Zhang, Ming Sun, Fengtong Wu

**Affiliations:** College of Computer and Control Engineering, Qiqihar University, Qiqihar 161003, China; 17862731836@163.com (B.H.); snogisunming@163.com (M.S.); a1273891327@163.com (F.W.)

**Keywords:** intelligent fault diagnosis, time series classification, convolutional neural networks

## Abstract

Compared to time-consuming and unreliable manual analysis, intelligent fault diagnosis techniques using deep learning models can improve the accuracy of intelligent fault diagnosis with their multi-layer nonlinear mapping capabilities. This paper proposes a model to perform fault diagnosis and classification by using a time series of vibration sensor data as the input. The model encodes the raw vibration signal into a two-dimensional image and performs feature extraction and classification by a deep convolutional neural network or improved capsule network. A fault diagnosis technique based on the Gramian Angular Field (GAF), the Markov Transition Field (MTF), and the Capsule Network is proposed. Experiments conducted on a bearing failure dataset from Case Western Reserve University investigated the impact of two coding methods and different network structures on the diagnosis accuracy. The results show that the GAF technique retains more complete fault characteristics, while the MTF technique contains a small number of fault characteristics but more dynamic characteristics. Therefore, the proposed method incorporates GAF images and MTF images as a dual-channel image input to the capsule network, enabling the network to obtain a more complete fault signature. Multiple sets of experiments were conducted on the bearing fault dataset at Case Western Reserve University, and the Capsule Network in the proposed model has an advantage over other convolutional neural networks and performs well in the comparison of fault diagnosis methods proposed by other researchers.

## 1. Introduction

In modern industry, machines are a key part of the production process. Failures of these machines can cause significant economic losses and sometimes pose a threat to the people who use them. In fact, more than 50% of mechanical defects are related to bearing failures [1,2]. Therefore, better and smarter bearing health-monitoring techniques are becoming an increasingly critical part of guaranteeing the proper and reliable operation of machines [3]. In order to obtain information about possible internal failures of a machine while it is working, one can only determine the internal state by analyzing the relevant external information. Through the literature on measurement science, the most useful and primary tool in the diagnosis of rolling bearing faults is the raw vibration signal [4,5].

Traditional fault diagnosis techniques usually fail when analyzing complex faults without further fault information. The intelligent diagnosis theory based on machine learning has recently become the core of research and application [6,7]. The intelligent diagnosis of bearing faults basically consists of two steps: Feature extraction and a classifier. Common signal processing techniques to extract representative features from the original signal include time-domain statistical analysis [8], wavelet transformation [9], and Fourier spectral analysis [10]. Moreover, common classifiers include k-nearest neighbor (KNN) [11], Multi-layer Perceptron (MLP) [12], support vector machine (SVM) [13], and so on. Based on the above technology, many researchers have conducted many research studies. In [14], a feature extraction method based on improved EMD energy entropy is analyzed and a genetic algorithm-based SVM is employed as a classifier. The research results prove that the intelligent feature extraction method outperforms manual annotation in both efficiency and effectiveness. Jia et al. found the problem of insufficient depth of ANNs and fed the spectrum into DNNs, achieving better results [15]. Guo et al. went on to construct a model using DNN as a classifier with multi-scale representations of time-domain, frequency-domain and time–frequency-domain features of the vibration signal [16].

In recent years, CNNs have also been used in fault diagnosis, due to their adaptive feature extraction capabilities. Convolutional neural networks or CNNs were first proposed by LeCun [17]. After this, more and more CNN architectures such as VGG-net [18] and Res-net [19] were proposed to play the role of extracting image features and a classifier. Although CNN is proposed for the classification task of images, its powerful feature extraction capability makes it suitable for the diagnosis of bearing fault features as well. Intuitively, the input for the CNN model for fault detection is one-dimensional raw time series data, which makes the feature extraction process shorter in duration, such as [20,21]. In 2021, Ozcan et al. went on to improve the 1D CNN for the purpose of fault diagnosis by enhancing the input fault features with multi-channel and multi-level inputs [22]. Furthermore, using 1D CNN, He et al. used Correlation Alignment (CORAL) to minimize the difference in the marginal distribution between the source and target domains [23]. However, it is much easier to extract information from data in a high dimension [24]. Another idea is the use of 2D CNN or 2D kernels, which allows the model to extract features in greater depth for better diagnostic results. Zhang et al. proposed a Convolution Neural Network with two dropout layers and two fully connected layers (DFCNN), and a two-dimensional grayscale image transformed by a one-dimensional vibrational signal was input in the DFCNN for training [25]. The model eliminates the effect of expert experience and shows good domain adaptation. Hoang et al. converted the vibration signals in the time domain into vibration images, which were then identified by VI-CNN classification [26]. Li et al. used WPT to obtain wavelet packet coefficients from vibration signals and convert them into two-dimensional arc images, which were then fed into the designed CNN model to achieve superior fault diagnosis capability [27]. The spectrogram of the vibration signal was used as the input to the CNN model by Pham et al. [28]. The model achieves high accuracy in diagnosing compound bearing faults under variable shaft speeds, and stability in noisy environments is demonstrated.

The above study demonstrates convolutional neural networks are a feasible solution to the fault diagnosis problem. CNNs are able to guarantee diagnostic efficiency without the experience of professionals and have a certain anti-noise ability in noisy environments. However, the input images of these schemes are derived from simple steps, allowing the performance of the entire scheme to be focused on the CNN model. However, CNNs have unavoidable drawbacks in fault diagnosis classification due to their inherent defect of translation invariance. After pointing out this question, Hinton proposed a solution—the Capsule Network [29]. Therefore, our study aims to make improvements in two areas: (1) Finding more complex encoding methods so that the generated 2D images have complete and nonlinear information about vibrational faults; and (2) trying to replace the CNN with a capsule network to ensure the network model has the possibility of higher accuracy. In certain coding methods of time series, Yang et al. selected the sensor time series and generated the coded map by coding and re-fusion of GAF and MTF, which essentially preserves the complete information of the original time series signal [30]. In fact, both GAF and MTF have been applied in many studies. For example, Mitiche et al. used the GAF technique to map the measured Electromagnetic Interference (EMI) time signals onto images from which important information was extracted [31]. Xiao et al. used GAF for feature extraction for hand motion classification of surface EMG. Bugueno et al. used a Markov transition field (MTF) to transform an unevenly sampled time series into a two-channel fixed-size image [32]. This image provides information to a convolutional neural network, which in turn classifies candidate transients. It is worth affirming that GAF and MTF techniques are feasible and effective.

In this paper, a new fault diagnosis method named GAFMTF-CapsNet is proposed. The original vibration signal is first encoded and converted into a two-dimensional grayscale image using GAF and MTF, and then the encoded image is input into a CNN or capsule network for training. During the experiment, we found that the GAF-encoded image preserves more complete static information, and the MTF-encoded image preserves the dynamic information of the original vibration signal. Therefore, the principle of GAFMTF-CapsNet is that the images obtained by these two conversion methods are fed as two channels into a Capsule Network with a small convolution kernel, so that this model can extract the static and dynamic characteristics of the original vibration signal with fault features. The excellent experimental performance of the model on the bearing dataset was investigated at Case Western Reserve University. The remainder of this paper is organized as follows: Section 2 presents the details of our proposed bearing fault diagnosis method, and Section 3 presents the specific process of three main experiments and a discussion of various experimental results. Section 4 presents the conclusions.

## 2. Methods

### 2.1. Transformation Method

With the rapid development of computer vision technology, the idea of using computer vision techniques to classify raw vibration signals has been inspired. The key is the conversion method to encode the vibration signal into a two-dimensional image (or array) acceptable to computer vision, and the process is required to reveal the features and patterns of the original vibration signal as much as possible.

The first transformation method is the Gramian Angular Field (GAF). The encoding of the time series into images by means of GAF polar coordinate-based matrices is able to maintain the correlation between the one-dimensional signal and the time series in Gramian matrices, where each element is actually the value of the trigonometric function of the angle [33]. The original time series x is first normalized to be between 0 and 1, which is defined in Equation (1).

(1) $${\tilde{x}}_{i}=\frac{x_{i}-x_{\min}}{x_{\max}-x_{\min}}$$

$x_{i}$ is the raw vibration signal at moment i and ${\tilde{x}}_{i}$ is the signal after normalization. $x_{\max}$ refers to the maximum value of x, and $x_{\min}$ is the minimum value of x. Then, we can use polar coordinates to represent the normalized time series $\tilde{x}$. The timestamp is the radius, and the formula is shown in Equation (2):

(2) $$\{\begin{array}{c} \phi =\arccos\left({\tilde{x}}_{i}\right),0\leq {\tilde{x}}_{i}\leq 1,{\tilde{x}}_{i}\in \tilde{X}\\ r=t_{i},t_{i}\in \mathbb{N} \end{array}$$

where t is the timestamp code at the moment. After converting the rescaled time series to the polar coordinate system, we can easily use the angular perspective to identify temporal correlations in different time intervals by considering the triangular sum/difference between each point. Using Gram’s Angular Sum Field (GASF) and Gram’s Angular Difference Field (GADF) encoded separately, the encoding is defined by Equations (3) and (4).

(3) $$\mathrm{GASF}=\left[\begin{array}{ccc} \cos\left(\phi_{1}+\phi_{1}\right) & \cdots & \cos\left(\phi_{1}+\phi_{n}\right)\\ \cos\left(\phi_{2}+\phi_{1}\right) & \cdots & \cos\left(\phi_{2}+\phi_{n}\right)\\ \vdots & \cos\left(\phi_{i}+\phi_{i}\right) & \vdots \\ \cos\left(\phi_{n}+\phi_{1}\right) & \cdots & \cos\left(\phi_{n}+\phi_{n}\right) \end{array}\right]$$

(4) $$\mathrm{GADF}=\left[\begin{array}{ccc} \cos\left(\phi_{1}-\phi_{1}\right) & \cdots & \cos\left(\phi_{1}-\phi_{n}\right)\\ \cos\left(\phi_{2}-\phi_{1}\right) & \cdots & \cos\left(\phi_{2}-\phi_{n}\right)\\ \vdots & \cos\left(\phi_{i}-\phi_{i}\right) & \vdots \\ \cos\left(\phi_{n}-\phi_{1}\right) & \cdots & \cos\left(\phi_{n}-\phi_{n}\right) \end{array}\right]$$

where $\phi_{i}$ denotes the angular value of the i-th sequence. It can be seen that after encoding in this way, the order on the two-dimensional image for the time from top left to bottom right is preserved. The original information is retained at the positive diagonal position, and the other regions express the relationship between different time sequences. For a vibration signal with an original time series length of n, a numerical matrix of n×n size is obtained by GAF encoding.

Another transformation method is the Markov Transition Field (MTF). The Markov transition field is a framework for encoding dynamic transition statistics, representing Markov transition probabilities sequentially to preserve information in the time domain [34]. For a given time series $X=\{x_{1},x_{2},x_{3}\ldots x_{n}\}$, their value domain is divided into Q intervals, then each $x_{i}$ can be mapped to a corresponding $q_{j}$. Thus, we can obtain a matrix W of Q×Q size. The element $w_{ij}$ of the matrix denotes the probability that an element in the interval j is followed by an element in the interval i and satisfies the condition $\sum_{j=1}^{Q}w_{ij}=1$. W is regarded as a Markov transition matrix. However, its dependence on the distribution and on time series X is not strong enough, and matrix W has too much information loss for the original time series. It has to be improved. The $M_{ij}$ cells in the Markov transition matrix M are defined by Equation (5).

(5) $$M_{ij}=\left[\begin{array}{ccc} w_{ij}|x_{1}\in q_{i},x_{1}\in q_{j} & \cdots & w_{ij}|x_{1}\in q_{i},x_{n}\in q_{j}\\ w_{ij}|x_{2}\in q_{i},x_{1}\in q_{j} & \cdots & w_{ij}|x_{2}\in q_{i},x_{n}\in q_{j}\\ \vdots & \ddots & \vdots \\ w_{ij}|x_{n}\in q_{i},x_{1}\in q_{j} & \cdots & w_{ij}|x_{n}\in q_{i},x_{n}\in q_{j} \end{array}\right]$$

In order to incorporate the temporal information into M, $M_{ij}$ is used to denote the transition probability from $q_{i}$ to $q_{j}$. The matrix M is defined by Equation (6).

(6) $$M=\left[\begin{array}{ccc} M_{11} & \cdots & M_{1n}\\ M_{21} & \cdots & M_{2n}\\ \vdots & \ddots & \vdots \\ M_{n1} & \cdots & M_{nn} \end{array}\right]$$

$M_{ij|\left|i-j\right|=k}$ denotes the transition matrix between the points with time interval k, and in M, the element $M_{ii}$ on the main diagonal is a special case of k=0, which denotes the probability of each interval to itself at time i. Finally, to ensure the matrix size is controllable, the fuzzy kernel is chosen to obtain the desired size.

### 2.2. Capsule Network

Capsule networks were proposed by Hinton in 2017 [29]. The units of a capsule network are called capsules, and each capsule contains multiple neurons. Unlike the scalar model of traditional convolutional neural networks, the capsules use vectors to label the positional relationships of the parts of the picture. The direction and length of the vector represent the probability of pointing to that direction, and a larger length indicates a higher probability. Usually, the capsule network contains two capsule layers, where the lower capsule makes predictions and provides parameter information to the higher capsule and activates the higher capsule when multiple predictions agree, and the activation function is “**squashing**” as shown in Equation (7):

(7) $$v_{j}=\frac{{\left\|s_{j}\right\|}^{2}}{1+{\left\|s_{j}\right\|}^{2}}\cdot \frac{s_{j}}{\left\|s_{j}\right\|}$$

where $v_{j}$ denotes the output of capsule j and $s_{j}$ denotes all the inputs of capsule j.

Since the capsule is a vector unit different from the neuronal scalar, the iterative update algorithm of the convolutional neural network will no longer be applicable. For the vector update, the capsule network uses the dynamic routing algorithm, which updates the weight parameter ${\vec{b}}_{ij}$ in the capsule by dynamic routing, ${\vec{b}}_{ij}$ is dotted by the prediction vector and the output vector, and the larger the dot product is, the greater the similarity between the prediction and the result. The specific way of updating the weights is as follows:

(1) The prediction vector ${\hat{u}}_{j|i}$, iteration number r, input capsule i, and output capsule j, parameter ${\vec{b}}_{ij}$.
(2) Update the vector $c_{ij}$ by Equation (8).
  (8) $$c_{ij}=\frac{exp(b_{ij})}{\sum_{k}exp(b_{ik})}$$
(3) The input capsule $s_{j}$ is obtained by Equation (9) and updated with the “squashing” (Equation (7)).
  (9) $$s_{j}=\sum_{i}c_{ij}{\hat{u}}_{j|i}$$
(4) Update ${\vec{b}}_{ij}$ by Equation (10) and return to step (2) for iterative calculation.
  (10) $$b_{ij}=b_{ij}+{\hat{u}}_{j|i}\cdot v_{j}$$
(5) Return the final output vector after r iterations.

Although the capsule network has an excellent feature extraction ability, it generates too many parameters at the input of large-size images, which greatly reduces the speed of network training. In order to reduce the number of parameters in the capsule network, improve the speed of the network, and ensure that not too much information is lost, a small-sized convolutional layer is constructed before the capsule layer to compress the image size and increase the depth to ensure the integrity of the input image information.

Because vectors in capsules are different from scalars, we use the length of the vector to represent the probability of positive correlation, so the model marginal loss function in this paper is the loss function, and each digital capsule k uses a separate marginal loss $L_{k}$ as shown in Equation (11):

(11) $$L_{k}=T_{k}\max{\left(0,m^{+}-\left\|v_{k}\right\|\right)}^{2}+\lambda \left(1-T_{k}\right)\max{\left(0,\left\|v_{k}\right\|-m^{-}\right)}^{2}$$

(12) $$Loss=\sum L_{k}$$

where $m^{+}=0.9$, $m^{-}=0.1$, $T_{k}$ is taken as 1 with the correct digital capsule, or 0. λ=0.5 aims to reduce the weight when wrong choices are made and reduce the compression of digital capsules by the training process. As in Equation (12), the total loss Loss is the sum of the individual digital capsule losses.

### 2.3. Proposed Methodology

The proposed fault diagnosis method has four main steps:

- Splitting. The original vibration signal is divided into appropriate signal segments to make it suitable for later encoding transformation. We will experiment to determine the appropriate signal split length.
- Encoding transformation. By the above transformation method, every segment is converted into a one-channel or two-channel image. The entire original vibration signal is converted into a large number of feature images. In this step, three transformation methods are considered feasible. There is GAF for the one-channel image, MTF for the one-channel image, and GAF and MTF for the two-channel image. We will compare the diagnostic accuracy of these methods in the experiment.
- Images datasets. The feature images are classified according to the fault type of the original vibration signal to form the image dataset.
- Network model. The image dataset is fed into the configured neural network model for training and testing. The image dataset will be fed into the conventional convolutional neural network and the proposed improved capsule network. We compare and summarize the advantages of the proposed network in the following experiments.

**Figure 1:**
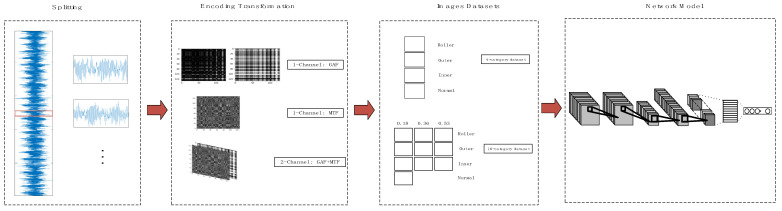
The whole process of fault diagnosis.

The whole fault diagnosis process is shown in Figure 1.

## 3. Experiments and Results

### 3.1. Dataset and Simulation Environment

**Figure 2:**
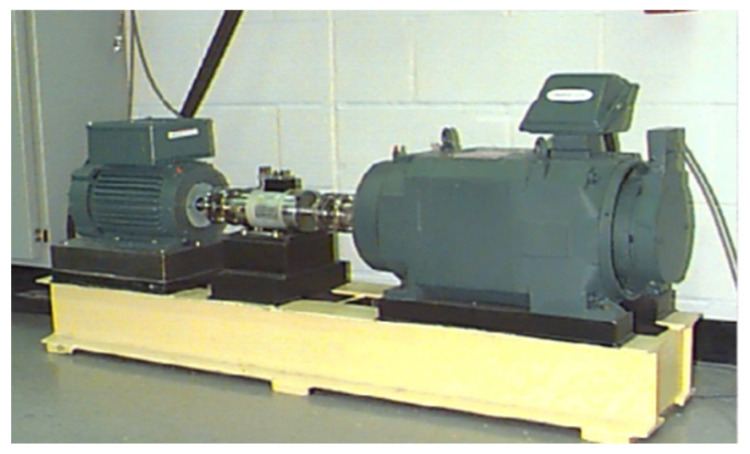
CWRU dataset experimental platform.

Our experiments were conducted using the Case Western Reserve University bearing dataset, which covers experimental drive-side bearing data sampled at 12 kHz and 48 kHz and 12 kHz fan-side bearing data. The experimental equipment included a 1.5 kw motor, a torque sensor/translator, and a power test meter [35]. The experimental platform is shown in Figure 2. The bearing failures were set to single-point damage from EDM. This dataset was designed with three damage types: Outer ring failure (OF), rolling element failure (RF), and inner ring failure (IF). For each type of damage, three levels of damage were set: 0.1778 mm, 0.3556 mm, and 0.5334 mm in diameter, plus a control experiment without failure (No), resulting in a total of 10 classified datasets, which were tested under four load conditions (0, 1, 2, and 3) with approximate motor speeds of 1797 r/min, 1772 r/min, 1750 r/min, and 1730 r/min. A total of 120 k sets of data were collected for each test set, and each set included drive-side acceleration data (DE), fan-side acceleration data (FE), and base acceleration data (BA). There are 120,000 sampling data points in all fault signal datasets and 240,000 sampling data points in the no-fault dataset. Drive-side acceleration data (DE) are adopted in this study.

The simulation tests are conducted by a personal computer with CPU (Intel(R) Core i7-10750H @ 2.60GHz) and GPU (NVIDIA GeForce RTX2070super 8G).

### 3.2. Experiment#1: GAF-Deeplearning

**Figure 3:**
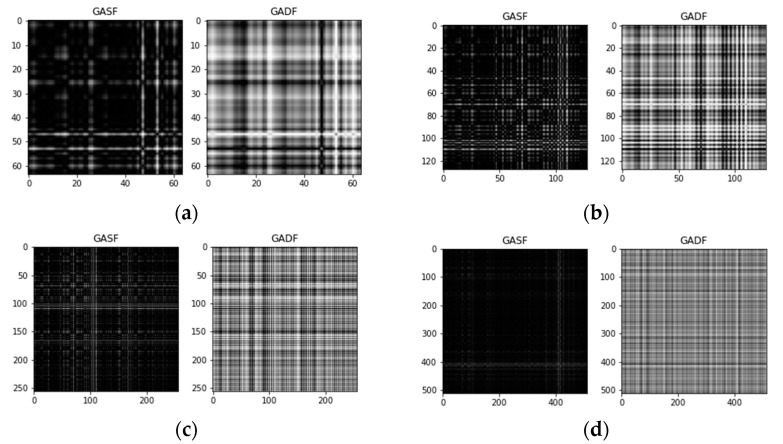
Different sizes of GAF images. (**a**) 64 × 64, (**b**) 128 × 128, (**c**) 256 × 256, (**d**) 512 × 512.

In the first experiment, we put GAF images into a capsule network or convolutional neural network model. First, we processed the raw vibration signal to make it easy to convert it into a GAF image. If you take a segment of the signal of length n and convert it in GAF, then you will obtain an n×n image (two-dimensional array). So, we need to cut and intercept long segments of signals in the dataset. However, too short an intercept length leads to incomplete fault features, which may make the training less accurate. We experimented with four sizes, 64, 128, 256, and 512, due to their more suitable input for neural networks. We made an average intercept of each fault type with a fixed length interval corresponding to its length. Each classification category is split into a training set and a test set according to a ratio of 8:2. In fact, the conversion ideas and algorithms of GASF and GADF are similar, but it is not certain that both ideas have the same results, so GASF and GADF are also tested separately. Figure 3 shows a schematic diagram of the GAF images for the four sizes. The GADF and GASF images are input into two network models.

**Figure 4:**
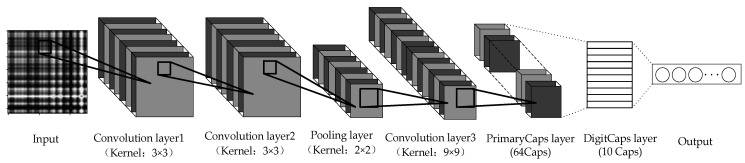
CapsNet model structure diagram.

The convolutional neural network was chosen to use the VGG16 network. The VGG16 network is a deep network model developed in 2014 by the Computer Vision Group at the University of Oxford together with researchers at Google DeepMind [18]. The network has a total of 16 training parameters. The VGG16 network achieved second place in the classification event and first place in the localization event of the ILSVRC 2014 competition. The VGGNet network has a simple structure and has very good generalization performance when migrating to other image data. VGGNet is still often used to extract image features. The capsule network structure suitable for fault diagnosis is constructed, including an input layer, a convolutional layer, a pooling layer, and a capsule layer. Due to the large size of the input GAF encoded image, in order to better extract features, the input is firstly passed through two layers of a 3 × 3 small kernel convolutional layer, compressed by one pooling layer, then passed through one layer of convolutional layer, and then input to the primary capsule layer, which has 64 capsules with 16 dimensions each, and then input to the digital capsule layer, which has 10 capsules corresponding to 10 types of faults. The CapsNet is shown in Figure 4.

**Figure 5:**
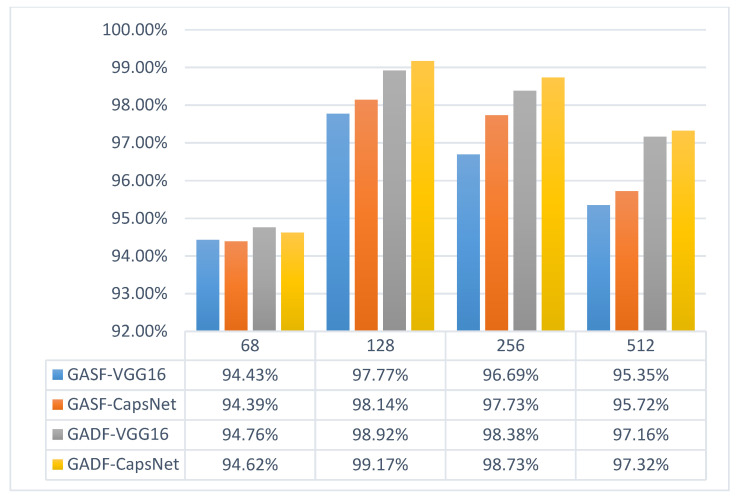
GAF-Deep learning experimental results.

Then, we input the encoded GADF and GASF images into VGG16 and CapsNet, respectively, for training. The experimental results are shown in Figure 5.

The experiment was summarized into the following three conclusions:

- When the size is 64, it is obvious that it is difficult to guarantee that an image contains complete information about the fault features. Images sized 128 have the best performance, and larger images contain more information but cause the network parameters to increase and make the training time of the network much longer.
- Except for the case of incomplete information at size 64, the experiments on images of other sizes show that GADF has higher accuracy than GASF.
- In the comparison between VGG and CapsNet, CapsNet performs better than VGG in most cases, but there is no significant advantage.

### 3.3. Experiment#2: MTF-Deeplearning

The second experiment uses MTF images input into the CapsNet and VGG16 networks. First, the original vibration signal is converted into an MTF image. The size of the MTF image is not fixed and is determined by both the length of the input signal and the size of the fuzzy kernel. The MTF technique embodies the dynamic characteristics of the original vibration, so the largest possible size of the signal is converted into an MTF image. However, too large an MTF input size can reduce the number of available training images or make the training set data too repetitive to extract fault features. Moreover, too large a fuzzy kernel size can reduce the MTF to the size we need, but this also causes information loss. For these two reasons, MTFs are required to have smaller fuzzy kernels and appropriate input vibration signal lengths.

**Figure 6:**
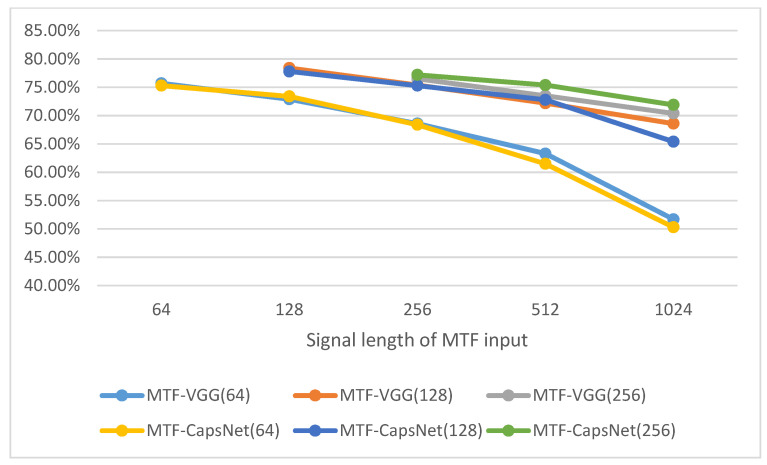
Accuracy of different MTF sizes.

However, in the actual experiments, we found that the experiments hardly showed satisfactory results regardless of the input length of the MTF images. Figure 6 shows the classification accuracy performance of the transformed images of vibration signals with different input lengths for VGG16 and CapsNet.

**Figure 7:**
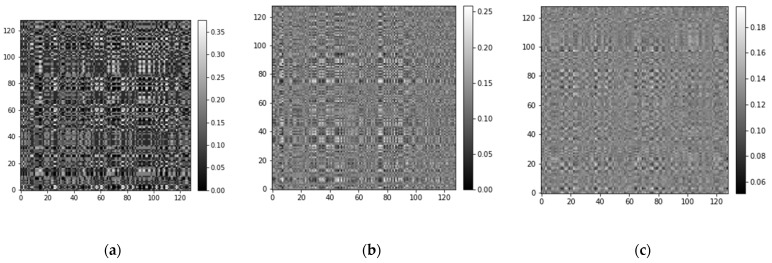
Different signal lengths are converted to 128 × 128 MTF images. (**a**) MTF image when the original signal length is 128. (**b**) MTF image when the original signal length is 256. (**c**) MTF image when the original signal length is 512.

In Figure 6, the horizontal axis is the length of the original vibration signal sampled corresponding to each MTF image. The numbers inside the brackets in the figure legend indicate the size of the MTF-converted 2D image. With this result, it can be seen that some information is lost in the MTF conversion process, resulting in a poorly trained network. The reason for this result may be that the encoding process of MTF focuses on the dynamic change process of the timing signal, leaving a large amount of dynamic information, while the static information is mostly lost. Nevertheless, MTF images can also extract the corresponding feature faults in the network training. In addition, when the MTF conversion input size is too large, the image becomes more blurred and thus fades out the fault features. As shown in Figure 7, we can obtain that conclusion intuitively.

It is concluded from Experiment 1 and Experiment 2 that the network model can extract different fault features from GAF images and MTF images, and that GAF retains much of the static information and MTF retains more dynamic information. Therefore, we consider a network model that makes it possible to extract fault features in GAF and MTF for the diagnosis and classification of faults separately. In addition, since the signal segmentation length that makes GAF work best is not the same as the signal segmentation length that makes MTF work best, we need to unify the two coded images by compromising one to the other.

### 3.4. Experiment#3: GAFMTF-Deeplearning

In this experiment, we want to let the deep learning model extract more complete fault features, so the model can extract fault features in both GAF images and MTF images. We encode the same vibration signal with both GAF and MTF, and the two images (arrays) after encoding are used as the two input channels of CapsNet. In this process, we need to change the CapsNet structure so that it takes a two-channel image as input. In addition, the size at which the GAF image appears best is different from the MTF image. We had to compromise the size of the MTF image based on the optimal size of the GAF image. We selected a size of 128 × 128 for this two-channel image. So, we need to split each signal in the fault dataset into vibration signals with lengths of 128. The processed images are divided into four 4-category datasets and four 10-category datasets, which are presented in Table 1 and Table 2. In this process, in order to avoid an imbalance in the number of data between different samples, the vibration signals in normal labels have different truncation intervals.

The converted image dataset is input into CapsNet for training, and the structure of CapsNet for 10-category and 4-category scenarios is shown in Table 3 and Table 4.

**Figure 8:**
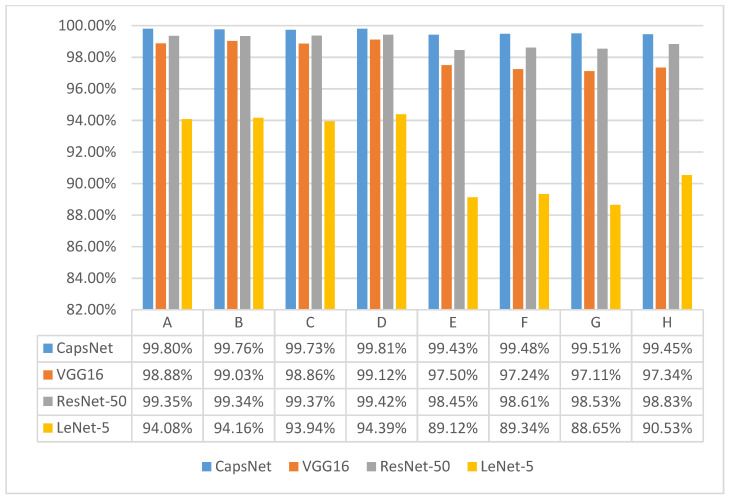
Accuracy of GAFMTF dataset in CapsNet and convolutional neural networks.

**Figure 9:**
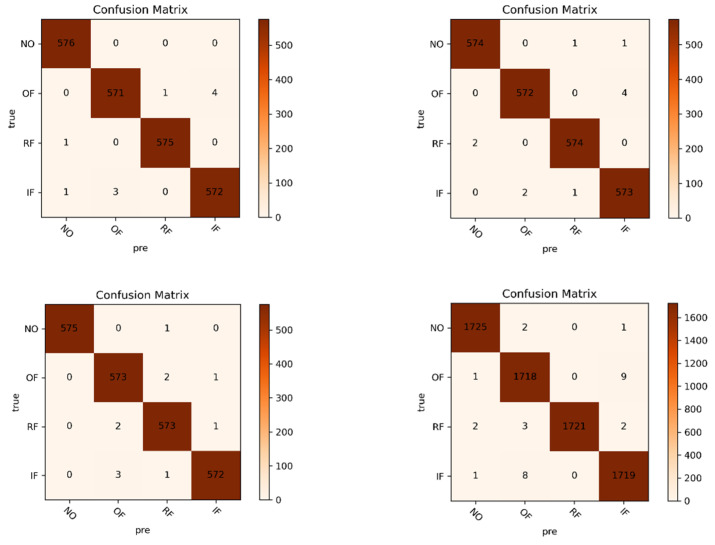

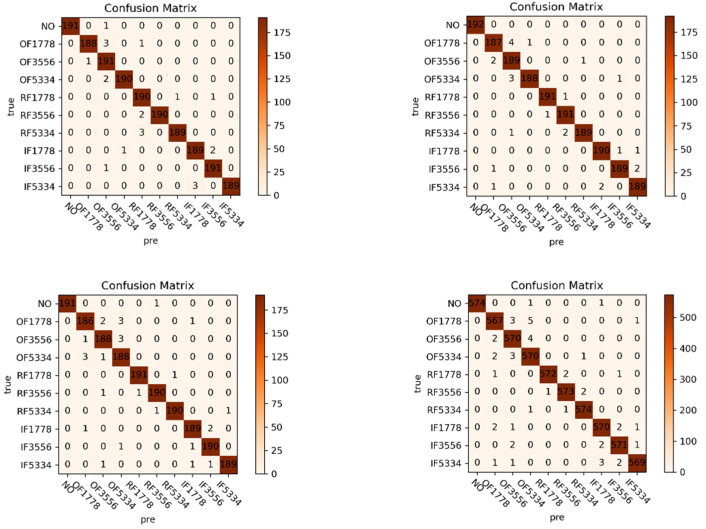
Confusion matrix of A-H eight datasets in CapsNet.

These eight datasets were trained on CapsNet and commonly used convolutional neural networks and compared. We experimented five times with cross-validation and averaged the test set accuracies of the five trials to produce Figure 8. In order to better investigate the advantages and disadvantages of the proposed method the confusion matrix is plotted to show the failure predictions of the network. Figure 9 shows the confusion matrix for the test set of eight datasets in GAFMTF-CapsNet.

In the figure, the eight confusion matrices correspond to the training results of eight datasets on CapsNet: ABCD is four-category datasets, where “NO” means no fault, “OF” means outer fault, “RF” means roller fault, and “IF” means inner fault. EFGH is 10-category datasets, where “NO, OF, RF, and IF” represent the fault location, and the number after the location is the degree of fault. We can see the failure diagnosis through the eight confusion matrices in Figure 9. Some interesting findings were found. CapsNet’s prediction of failure or non-failure is accurate and clear. In the experiments, few no-fault images were predicted as faulty. Most of the failures were predicted to be a worry fault of the correct location, and the outer fault was more likely to be incorrectly predicted.

Sometimes the number of datasets does not necessarily mean the network is adequately trained. The datasets of the previous experiments were in a ratio of 8:2 for the training set and the test set. We re-trained with dataset D and dataset H to explore the performance with a smaller training dataset. In this experiment, we re-divided dataset D and dataset H. CapsNet was retrained inputting the new dataset, which had ratios of 6:4, 7:3, 8:2, and 9:1 for the training set with the test set. We performed multiple trials to reduce errors by cross-validation. The average accuracy is shown in Table 5.

We find that for a four-category dataset, reducing the training set does not lead to a significant decrease in accuracy. Even 60% of the training set is enough for CapsNet to extract fault features. For the 10-category dataset, 70% of the training set can still obtain good diagnostic accuracy. Fewer training episodes would not allow CapsNet to extract enough fault features and thus the accuracy would be significantly reduced.

Through the results, it seems that CapsNet has higher accuracy than other convolutional neural networks. However, there is no significant advantage of GAFMTF-CapsNet in terms of accuracy when compared with the methods for fault diagnosis in other studies, which are presented in Table 6.

Although the proposed method is not the best, the GAF and MTF transformations are proved to be feasible for fault feature extraction of bearings.

## 4. Conclusions

In this paper, the application of GAF and MTF techniques in bearing fault diagnosis is studied and a feasible fault diagnosis method (GAFMTF-CapsNet) is proposed. In the experiment, we obtained the following conclusions:

- The GAF technique retains sufficient bearing failure characteristics. Neural networks more easily extract fault features and perform classification and diagnosis by GAF images. For the capsule network and VGG network, the original vibration signal cut into 128 and converted into a 128 × 128 GAF image has a better performance compared to other cut lengths. GAF-CapsNet has, at most, 99.17% accuracy in training 128 × 128 GAF images.
- For different sizes of signal cuts and output image sizes, MTF is only less than 80% accurate in CapsNet and VGG. The MTF technique loses a large number of fault characteristics, but also retains some of them. MTF retains more dynamic features and less static information than the GAF technique.
- The four-category dataset and the 10-category dataset have an average accuracy of 99.8% and 99.5% on GAFMTF-CapsNet. Using GAF and MTF images as two-channel inputs in the neural network allows the network to be trained to obtain both the static information by GAF and dynamic information by MTF for the vibration signal. For this signal processing method (GAFMTF), CapsNet has 1–2% higher diagnostic accuracy than VGG16 and ResNet-50, and 5–10% higher accuracy than LeNet-5.

Compared with other fault diagnosis methods based on deep learning, the proposed GAFMTF-CapsNet has higher diagnostic accuracy. In addition, the proposed method does not require any a priori knowledge, so it can be applied more widely. However, the use of GAFMTF-CapsNet requires the advance acquisition of the operating signals of the same bearing in the fault condition. Moreover, the raw vibration signal may be inaccurate. These issues will be investigated later to further improve the method.

## Figures and Tables

**Table 1:** GAFMTF four-category datasets.

Fault Location		None	Inner	Roller	Outer	Load
Label		1	2	3	4	
A	train	2304	2304	2304	2304	1
	test	576	576	576	576	
B	train	2304	2304	2304	2304	2
	test	576	576	576	576	
C	train	2304	2304	2304	2304	3
	test	576	576	576	576	
D	train	6912	6912	6912	6912	1,2,3
	test	1728	1728	1728	1728	

**Table 2:** GAFMTF 10-category datasets.

Fault Location		None	Inner			Roller			Outer			Load
Label		1	2	3	4	5	6	7	8	9	10	
Diameter		0	0.1778	0.3556	0.5334	0.1778	0.3556	0.5334	0.1778	0.3556	0.5334	
E	train	768	768	768	768	768	768	768	768	768	768	1
	test	192	192	192	192	192	192	192	192	192	192	
F	train	768	768	768	768	768	768	768	768	768	768	2
	test	192	192	192	192	192	192	192	192	192	192	
G	train	768	768	768	768	768	768	768	768	768	768	3
	test	192	192	192	192	192	192	192	192	192	192	
H	train	2304	2304	2304	2304	2304	2304	2304	2304	2304	2304	1,2,3
	test	576	576	576	576	576	576	576	576	576	576	

**Table 3:** CapsNet for 10-category layer architecture.

Layer Type	Kernel Size /Caps Dimension	Kernel Channel /Caps Number	Padding	Output
Input				128 × 128 × 2
Convolution 1	3 × 3	64	Yes	128 × 128 × 128
Convolution 2	3 × 3	128	Yes	128 × 128 × 128
Pooling	2 × 2	128	No	64 × 64 × 128
Convolution 3	9 × 9	256	Yes	64 × 64 × 256
PrimaryCaps	16	64		32 × 32 × 16 × 64
DigitCaps	16	10		16 × 10
Output				10

**Table 4:** CapsNet for 4-category layer architecture.

Layer Type	Kernel Size /Caps Dimension	Kernel Channel /Caps Number	Padding	Output
Input				128 × 128 × 2
Convolution 1	3 × 3	64	Yes	128 × 128 × 128
Convolution 2	3 × 3	128	Yes	128 × 128 × 128
Pooling	2 × 2	128	No	64 × 64 × 128
Convolution 3	9 × 9	256	Yes	64 × 64 × 256
PrimaryCaps	16	64		32 × 32 × 16 × 64
DigitCaps	16	4		16 × 4
Output				4

**Table 5:** Accuracy at different numbers of training sets.

Datasets	6:4	7:3	8:2	9:1
D (4-category)	96.54%	98.77%	99.81%	99.82%
H (10-category)	92.39%	97.21%	99.45%	99.43%

**Table 6:** Bearing fault diagnosis by GAFMTF-CapsNet and other methods.

Methods	Researchers	Categories	Accuracy
GAFMTF-CapsNet		4	99.81%
		10	99.51%
Spark-IRFA	Wan, L. J. [36]	4	98.12%
VI-CNN	Hoang, D. T. [26]	4	100%
STFT-CNN	Pham, M. T. [28]	4	99.4%
WPT-CNN	Li, G. Q. [27]	6	99.44%
Improved 2D LeNet-5 network	Wan, L. J. [37]	10	99.25%
Improved 1D LeNet-5 network	Wan, L. J. [37]	10	99.66%
VCN	Wang, Y. J. [38]	10	99.53
DFCNN	Zhang, J. Q. [25]	10	100%

## Data Availability

Case Western Reserve University Bearing Data https://engineering.case.edu/bearingdatacenter.

## References

[1] Wang W., Lee H. (2013). An energy kurtosis demodulation technique for signal denoising and bearing fault detection. Meas. Sci. Technol..

[2] Yiakopoulos C.T., Gryllias K.C., Antoniadis I.A. (2011). Rolling element bearing fault detection in industrial environments based on a K-means clustering approach. Expert Syst. Appl..

[3] Fong A.C.M., Hui S.C. (2001). An intelligent online machine fault diagnosis system. Comput. Control Eng. J..

[4] Liu Y., Zhang J.H., Bi F.R., Lin J.W., Ma W.P. (2015). A fault diagnosis approach for diesel engine valve train based on improved ITD and SDAG-RVM. Meas Sci. Technol..

[5] Chen B.J., He Z.J., Chen X.F., Cao H.R., Cai G.G., Zi Y.Y. (2011). A demodulating approach based on local mean decomposition and its applications in mechanical fault diagnosis. Meas. Sci. Technol..

[6] Lei Y.G., He Z.J., Zi Y.Y. (2009). Application of an intelligent classification method to mechanical fault diagnosis. Expert Syst. Appl..

[7] Wang D., Tse P.W., Guo W., Miao Q.A. (2011). Support vector data description for fusion of multiple health indicators for enhancing gearbox fault diagnosis and prognosis. Meas Sci. Technol..

[8] Wang X., Zheng Y., Zhao Z.Z., Wang J.P. (2015). Bearing Fault Diagnosis Based on Statistical Locally Linear Embedding. Sensors.

[9] Li K., Chen P., Wang S.M. (2012). An Intelligent Diagnosis Method for Rotating Machinery Using Least Squares Mapping and a Fuzzy Neural Network. Sensors.

[10] Rai V.K., Mohanty A.R. (2007). Bearing fault diagnosis using FFT of intrinsic mode functions in Hilbert-Huang transform. Mech. Syst. Signal Process..

[11] Pandya D.H., Upadhyay S.H., Harsha S.P. (2013). Fault diagnosis of rolling element bearing with intrinsic mode function of acoustic emission data using APF-KNN. Expert Syst. Appl..

[12] Li B., Chow M.Y., Tipsuwan Y., Hung J.C. (2000). Neural-network-based motor rolling bearing fault diagnosis. IEEE Trans. Ind. Electron..

[13] Santos P., Villa L.F., Renones A., Bustillo A., Maudes J. (2015). An SVM-Based Solution for Fault Detection in Wind Turbines. Sensors.

[14] Huang J., Hu X.G., Yang F. (2011). Support vector machine with genetic algorithm for machinery fault diagnosis of high voltage circuit breaker. Measurement.

[15] Jia F., Lei Y.G., Lin J., Zhou X., Lu N. (2016). Deep neural networks: A promising tool for fault characteristic mining and intelligent diagnosis of rotating machinery with massive data. Mech. Syst. Signal Process..

[16] Guo L., Gao H.L., Huang H.F., He X., Li S.C. (2016). Multifeatures Fusion and Nonlinear Dimension Reduction for Intelligent Bearing Condition Monitoring. Shock. Vib..

[17] Lecun Y., Bottou L., Bengio Y., Haffner P. (1998). Gradient-based learning applied to document recognition. Proc. IEEE.

[18] Simonyan K., Zisserman A.J. (2014). Very Deep Convolutional Networks for Large-Scale Image Recognition. arXiv.

[19] He K., Ren X.Z.S., Sun J. Deep residual learning for image recognition. Proceedings of the IEEE Conference on Computer Vision and Pattern Recognition.

[20] Ince T. (2019). Real-time broken rotor bar fault detection and classification by shallow 1D convolutional neural networks. Electr. Eng..

[21] Abdeljaber O., Avci O., Kiranyaz S., Gabbouj M., Inman D.J. (2017). Real-time vibration-based structural damage detection using one-dimensional convolutional neural networks. J. Sound Vib..

[22] Ozcan I.H., Devecioglu O.C., Ince T., Eren L., Askar M. (2021). Enhanced bearing fault detection using multichannel, multilevel 1D CNN classifier. Electr. Eng..

[23] He J., Li X., Chen Y., Chen D.F., Guo J., Zhou Y. (2021). Deep Transfer Learning Method Based on 1D-CNN for Bearing Fault Diagnosis. Shock. Vib..

[24] Ding X.X., He Q.B. (2017). Energy-Fluctuated Multiscale Feature Learning With Deep ConvNet for Intelligent Spindle Bearing Fault Diagnosis. IEEE Trans. Instrum. Meas..

[25] Zhang J.Q., Sun Y., Guo L., Gao H.L., Hong X., Song H.L. (2020). A new bearing fault diagnosis method based on modified convolutional neural networks. Chin. J. Aeronaut..

[26] Hoang D.T., Kang H.J. (2019). Rolling element bearing fault diagnosis using convolutional neural network and vibration image. Cogn. Syst. Res..

[27] Li G.Q., Deng C., Wu J., Chen Z.Y., Xu X.B. (2020). Rolling Bearing Fault Diagnosis Based on Wavelet Packet Transform and Convolutional Neural Network. Appl. Sci..

[28] Pham M.T., Kim J.M., Kim C.H. (2020). Accurate Bearing Fault Diagnosis under Variable Shaft Speed using Convolutional Neural Networks and Vibration Spectrogram. Appl. Sci..

[29] Sabour S., Frosst N., Hinton G.E. (2017). Dynamic Routing Between Capsules. NIPS’17: Proceedings of the 31st International Conference on Neural Information Processing, Long Beach, CA, USA, 4–9 December 2017.

[30] Yang C.L., Chen Z.X., Yang C.Y. (2020). Sensor Classification Using Convolutional Neural Network by Encoding Multivariate Time Series as Two-Dimensional Colored Images. Sensors.

[31] Mitiche I., Morison G., Nesbitt A., Hughes-Narborough M., Stewart B.G., Boreha P. (2018). Imaging Time Series for the Classification of EMI Discharge Sources. Sensors.

[32] Bugueno M., Molina G., Mena F., Olivares P., Araya M. (2021). Harnessing the power of CNNs for unevenly-sampled light-curves using Markov Transition Field. Astron. Comput..

[33] Oates Z.W.T. Imaging Time-Series to Improve Classification and Imputation. Proceedings of the Twenty-Fourth International Joint Conference on Artificial Intelligence.

[34] Oates Z.W.T. Encoding Time Series as Images for Visual Inspection and Classification Using Tiled Convolutional Neural Networks. Proceedings of the Workshops at the Twenty-Ninth AAAI Conference on Artificial Intelligence.

[35] Loparo K. (2003). Bearing vibration Data Set.

[36] Wan L.J., Gong K., Zhang G., Yuan X.P., Li C.Y., Deng X.J. (2021). An Efficient Rolling Bearing Fault Diagnosis Method Based on Spark and Improved Random Forest Algorithm. IEEE Access.

[37] Wan L.J., Chen Y.W., Li H.Y., Li C.Y. (2020). Rolling-Element Bearing Fault Diagnosis Using Improved LeNet-5 Network. Sensors.

[38] Wang Y.J., Ding X.X., Zeng Q., Wang L.M., Shao Y.M. (2021). Intelligent Rolling Bearing Fault Diagnosis via Vision ConvNet. IEEE Sens. J..

